# Ultrastructure imaging of *Pseudomonas aeruginosa* lawn biofilms and eradication of the tobramycin-resistant variants under *in vitro* electroceutical treatment

**DOI:** 10.1038/s41598-020-66823-y

**Published:** 2020-06-18

**Authors:** Varun Lochab, Travis H. Jones, Devendra H. Dusane, Casey W. Peters, Paul Stoodley, Daniel J. Wozniak, Vish V. Subramaniam, Shaurya Prakash

**Affiliations:** 1 Department of Mechanical & Aerospace Engineering, The Ohio State University, Columbus Ohio, USA; 2 Department of Microbial Infection & Immunity, The Ohio State University, Columbus Ohio, USA; 3 Department of Orthopaedics, The Ohio State University, Columbus Ohio, USA; 4 National Centre for Advanced Tribology, Mechanical Engineering, University of Southampton, Southampton, UK

**Keywords:** Microbiology, Biofilms, Mechanical engineering

## Abstract

Electrochemically generated bactericidal compounds have been shown to eradicate bacterial lawn biofilms through electroceutical treatment. However, the ultrastructure of biofilms exposed to these species has not been studied. Moreover, it is unknown if the efficacy of electroceutical treatment extends to antibiotic-resistant variants that emerge in lawn biofilms after antibiotic treatment. In this report, the efficacy of the *in vitro* electroceutical treatment of *Pseudomonas aeruginosa* biofilms is demonstrated both at room temperature and in an incubator, with a ~4 log decrease (p < 0.01) in the biofilm viability observed over the anode at both conditions. The ultrastructure changes in the lawn biofilms imaged using transmission electron microscopy demonstrate significant bacterial cell damage over the anode after 24 h of electroceutical treatment. A mix of both damaged and undamaged cells was observed over the cathode. Finally, both eradication and prevention of the emergence of tobramycin-resistant variants were demonstrated by combining antibiotic treatment with electroceutical treatment on the lawn biofilms.

## Introduction

Biofilms are structured aggregates of bacteria embedded in self-produced extracellular polymeric substances (EPS) including proteins, DNA, and polysaccharides^1,2^. Biofilms on or embedded in living tissues, such as wounds, can cause chronic infections that may be resistant to killing by systemic antibiotics, topical antiseptics, and the host immune system^3,4^. Biofilms usually present a persistent pathology^4–6^. For instance, chronically infected wounds are generally non-healing^3,6^ and affect over 6.5 million individuals in the US alone with an estimated ~$25 billion spent annually on healthcare costs^6^.

We recently reported on the screen-printed silver/silver chloride (Ag/AgCl) wound dressings called electroceuticals^7^, which are conformable to the wound morphology and present an emerging paradigm for the treatment of open, chronic, and biofilm-infected wounds. Past work has shown that electrochemical treatment by electroceuticals eradicates or inhibits the growth of both planktonic bacteria and bacterial biofilms^8–12^. However, the use of electroceuticals under different treatment conditions and the impact of these electrochemical treatments on the biofilm ultrastructure, which is defined as the detailed structure of bacterial cells including sub-cellular structures and the structure of the cell envelope, is not well understood. Furthermore, it is unknown if the treatment efficacy of electrochemically generated bactericidal species extends to the antibiotic-resistant variants that have been shown to emerge after standard antibiotic treatment of biofilms^13^.

In this paper, we address these knowledge gaps using *in vitro* electrochemical treatment on *Pseudomonas aeruginosa* (PA) lawn biofilms and the emergent antibiotic-resistant variants. Notably, PA is one of the most common nosocomial pathogens isolated from chronic wound infections^14,15^. PA infections are treated using clinically relevant antibiotics such as tobramycin^16,17^. However, PA exhibits resistance to multiple antimicrobial agents due to its genome that encodes for antibiotic inactivating enzymes and multi-drug efflux pumps^15,18^. PA biofilms express drug-tolerant persister cells and drug-resistant variants that can survive aggressive antibiotic treatments, host immune defenses, and present a persistent pathology^14,15^. Moreover, the antibiotic resistance in a biofilm can be 1000-fold larger than the planktonic bacteria^19,20^.

Previous *in vitro* work on the electrochemical treatment of bacteria has shown that bacterial eradication spans a large range of electrode materials — silver (Ag), platinum (Pt), gold (Au), stainless steel, and copper (Cu)^8^. The bacterial eradication was observed over a broad direct current (dc) range, reported as current density, from ~ 0.02 to 20.00 µA/mm^2^ for treatment of *Staphylococcus aureus*, *Escherichia coli*, *Proteus vulgaris, and Pseudomonas aeruginosa* (PA)^8^. At the highest current density of ~20.00 µA/mm^2^, all electrode materials inhibited bacterial growth at both the anode and the cathode^8^; however, Ag anodes also showed inhibition at the lowest current density levels of 0.02 µA/mm^2^ with minimal inhibition at the Ag cathodes. Furthermore, electrochemical treatments *in vivo* of PA infected wounds in rabbits with Cu mesh electrodes also exhibited infection clearance^21^.

Mechanistically, for Pt electrodes, electrochemically generated hypochlorous acid (HOCl), which is a reactive chlorine species (RCS)^22^, was considered responsible for bactericidal effects observed on *Staphylococcus epidermidis* biofilms grown on polycarbonate disks under physiological saline conditions^23^. Further, cathodic polarization of Pt coupons (−1.5 V and −1.8 V vs. Ag/AgCl) incubated in cultures of methicillin-resistant *Staphylococcus aureus* and *Acinetobacter baumannii* showed reduced bacterial attachment and viability^24^. Sultana *et. al*. demonstrated that electrochemically generated hydrogen peroxide (H_2_O_2_), a reactive oxygen species (ROS)^22^, increased the tobramycin susceptibility in PA biofilms^11^. The electrochemical scaffold (or e-scaffold) made with a conductive carbon fabric^11^ was polarized at a constant potential against standard Ag/AgCl reference electrodes for production of H_2_O_2_. The e-scaffold was also recently used to generate HOCl for the treatment of *in vitro* biofilms^25^. Dusane *et al*.^26^ reported an innovative soft-tissue infection *in vitro* assay. In their work, Dusane *et al*. showed the electroceutical eradication of a 24-hour grown lawn biofilm of bioluminescent *Pseudomonas aeruginosa* Xen 41 (PA-Xen41) using Ag electrodes embedded in nutrient agar. HOCl and other derived reaction products such as chloramines were reported as the likely species for observed bactericidal effects^26^.

Despite growing evidence for ROS and RCS being the anti-bacterial agents in the electrochemical treatment of biofilms, the effect of these bactericidal species on biofilm ultrastructure at any of the electrical poles i.e., the anode or the cathode is unknown. Furthermore, there are no systematic reports with a reliable investigation of the treatment efficacy of electrochemically generated bactericidal species at different treatment conditions, including when the antibiotic-resistant variants emerge from a lawn biofilm after the application of a standard antibiotic treatment^13^.

In this report, the electroceutical treatment of PA-Xen41 lawn biofilms was assessed by time-lapse quantification of biofilm viability and bacterial luciferase activity maps, both at room temperature (~25 °C) under ambient laboratory conditions, and in an incubator (temperature, T = 37 °C and 5% CO_2_ concentration). Next, the effects of electrochemically generated bactericidal species on the ultrastructure of the lawn biofilms were observed using transmission electron microscopy (TEM) for both the anode and cathode at select time intervals over a 24-hour treatment period. Finally, we evaluated the combined antibiotic and electroceutical treatment of tobramycin-resistant phenotypes that emerge from the PA lawn biofilms after standard tobramycin treatment.

## Methods

### Agar based tissue model and quantification of biofilm viability

**Figure 1:**
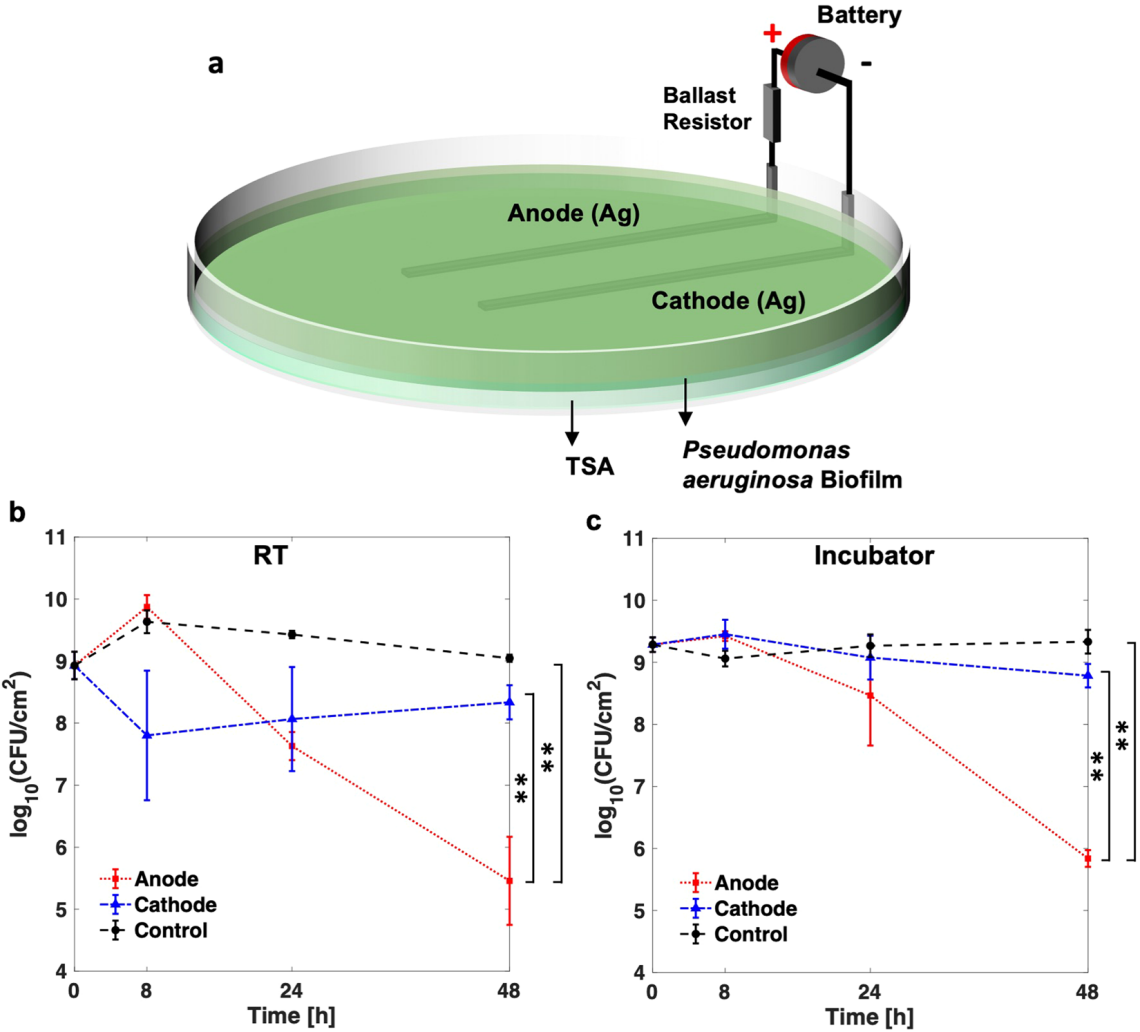
(**a**) Schematic for the electroceutical treatment^26^ of bioluminescent *Pseudomonas aeruginosa* (PA-Xen41) biofilm. Quantification of PA-Xen41 lawn biofilm viability in colony-forming units per unit area (CFU/cm^2^) for electroceutical treatment at **(b)** RT and **(c)** in the incubator (37 °C and 5% CO_2_) as a function of time (in hours). The ** denotes a p-value ≤ 0.01.

A bioluminescent strain of *Pseudomonas aeruginosa* (or PA-Xen41) (PerkinElmer, USA) was used in all experiments with culture methods and conditions reported previously^26^. The development and use of the agar-based *in vitro* tissue model (Fig. 1a) for electroceutical treatment of the lawn biofilms was also recently reported^26^. Briefly, sterilized Ag foil electrodes (0.1 mm thick, 99.9% purity, Sigma-Aldrich, USA) were placed in a petri dish (150 mm outer diameter, Fisher Scientific, USA). The Ag electrodes were 80 mm long × 3 mm wide with 3 cm spacing between the electrodes. Sterilized 55 mL tryptic soy agar (TSA), containing 1.5% (w/v) agar (Fisher Scientific, USA) prepared with 3% (w/v) tryptic soy broth (TSB, Sigma Aldrich, USA) was poured over the electrodes and cooled aseptically to form a gel (≈3.6 mm thick) at room temperature (RT). Next, 100 µL of an overnight culture of PA-Xen41 grown in TSB was diluted with 9.9 mL TSB to prepare the inoculum. 400 µL of this inoculum was spread on the surface of the TSA gel using a sterile glass spreader. The petri dishes were then cultured for 24 h at 37 °C and 5% CO_2_ in a humidified incubator (Heracell 150i, Thermo Scientific)^26^. It has been previously shown that these bacterial lawns exhibit phenotypic characteristics of biofilms, and therefore, they are typically referred to as lawn biofilms^26,27^.

The electroceutical treatment of PA-Xen41 lawn biofilms (Fig. 1a) was started after 24 h of biofilm growth marked as *t* = 0 h, where *t* is the treatment time. The electroceutical treatment was applied i.e., current was ‘on’ until *t* = 24 h. As previously reported^26^, a portable battery (two 3 V Lithium cells in series, CR2032 Energizer) with a nominal voltage of 6 V was used as a DC power source and a ballast resistor of 1 kΩ was used to limit the current flow. The current at the beginning of the treatment was typically ~3.5 mA and reduces to ~100 μA at *t* = 24 h with the growth of AgCl layer on the anode, while complete experimental details were reported previously^26^. The protocols for obtaining colony-forming units and bacterial luciferase activity maps through *in vivo* imaging system or IVIS (Xenogen IVIS 100, Hopkinton, USA) imaging have been reported previously^26^. Briefly, IVIS imaging is used as a large field of view, with length scales on the order of 10 cm, optical imaging modality for qualitative monitoring of the metabolic activity and the growth of bioluminescent reporters^26,28^. PA-Xen 41 expresses luciferase bioluminescence, which is captured by Xenogen IVIS 100 using a back-illuminated charged coupled device (CCD) camera.

### pH measurement

Quantitative pH measurements at ~2 mm immersion depth from the surface of the TSA using a pH microelectrode per vendor specifications (Microelectrodes Inc., USA) are reported. The pH measurements were directly over anode, cathode, and at least 2 cm away from both electrodes to serve as an internal control. pH measurements from three different sets of experiments and mean ± standard deviations are reported.

### Imaging biofilm microstructure and ultrastructure

Transmission Electron Microscopy was used to assess changes in bacterial cell ultrastructure due to the electroceutical treatment. Lawn biofilm samples on agar were obtained by using a 3 mm diameter punch from the anode, cathode, and control (i.e., from lawn biofilms where electroceutical treatment was not applied). These plugs were also used for quantifying viable cells as colony-forming units (CFUs). The punched samples (i.e., biofilm on ≈ 3.6 mm underlying TSA) were fixed in 2.5% glutaraldehyde (25% EM grade, #18426 Ted Pella, Inc.) in 0.1 M phosphate buffer solution (PBS at pH = 7.4) overnight at 4 °C. After fixation, the samples were rinsed twice for ten minutes each in 0.1 M PBS, and subsequently, stained with 1% osmium tetroxide (#18456 Ted Pella, Inc.) in 0.1 M PBS for 2 h at RT. The samples were then rinsed twice for five minutes each in water and then stained in 1% uranyl acetate (ACS grade, #19481, Ted Pella, Inc.) for 1 h at RT. Next, the samples were dehydrated in a graded ethanol (190 proof, #2805HC Decon Labs, Inc.) series diluted with distilled water for the following concentration sequence: 50% ×2, 70% ×2, 80% ×2, 95% ×2 with each dehydration step being 10 minutes long. The dehydration step was completed with the final two changes of 100% ethanol (200 proof) for 15 minutes each and two changes of acetone (HPLC Grade, #A949-4 Fisher Scientific) for 15 minutes each. The samples were infiltrated with a series of Eponate 12 (Ted Pella, Inc.) epoxy resin and acetone according to the following scheduled steps: (a) 1:1 of resin mix: acetone for 2 h, (b) 2:1 resin mix: acetone for 3 h, (c) 100% resin mix for 3 h, (d) another 100% resin mix for 3 h. The samples were then moved to freshly made resin mix in a silicone mold and the resin was cured in an oven at 60 °C for 48 h. The resin blocks with embedded gel and biofilm samples were then manually trimmed with a razor blade, and the samples were sectioned on an ultramicrotome (Leica UC6) using a diamond knife (Diatome) with a 6° clearance angle in 90 nm thick sections. Finally, 90 nm thick sections were stained with 1% aqueous uranyl acetate for 3 minutes followed by 2 minutes in lead citrate (EM Grade, #19314 Ted Pella, Inc.). The samples were observed using FEI Tecnai G2 Spirit TEM at 80 kV. The TEM images were captured with an Advanced Microscopy Techniques camera.

### Combining antibiotic and electroceutical treatments

To expose the lawn biofilms to a combination of antibiotic and electroceutical treatments, a modified Kirby-Bauer method was used^13^. In this method, blank paper disks (6 mm diameter, Remel Blank Paper Disk, Thermo Fisher Scientific) loaded with 100 µg tobramycin (TOB), delivered in a 10 µL drop and allowed to dry, were used. Three such antibiotic-loaded disks were placed over 24 h PA-Xen41 lawn biofilms. The disks were equidistant from each other and the periphery of the petri dish (140 mm inner diameter). The positions of the disks (one at the center of the petri dish and two placed at 35 mm away from the center disk on each side, i.e., towards the anode and the cathode) were marked using a Vernier caliper on the petri dishes before the start of the experiments (Fig. S1). Two separate experiments were performed after the placement of TOB loaded disks (marked as time, *t* = 0 h): one for observing the effects of electroceutical treatment on TOB-resistant variants which grow in the cleared zone after extended incubations in the presence of antibiotics^29^ and the other for evaluating if the electroceutical treatment can prevent the emergence of TOB-resistant variants.

#### Treatment of antibiotic-resistant variants in PA-Xen41 lawn biofilms

TOB loaded paper disks were placed over the 24 h grown PA-Xen41 lawn biofilm at *t* = 0 h, which was incubated at 37 °C and 5% CO_2_ until *t* = 72 h. The antibiotic-resistant phenotypes emerge in the cleared lawn at *t* = 72 h in IVIS imaging^13^. After emergence of the antibiotic-resistant phenotypes, electroceutical treatment was applied for an additional 24 h (i.e., from *t* = 72 h to *t* = 96 h). The response of the antibiotic-resistant phenotypes to treatment was observed using both IVIS and white light digital imaging.

#### Prevention of the emergence of antibiotic-resistant variants in PA-Xen41 lawn biofilms

The TOB loaded paper disks were placed on top of the lawn biofilms and the electroceutical treatment was started immediately (i.e., *t* = 0 h) and continued for 24 h in the 37 °C incubator. After the electroceutical treatment was stopped at *t* = 24 h, the culture plates continued to be incubated until *t* = 72 h. IVIS images were recorded every 24 h to observe the metabolic activity maps of PA-Xen41 lawn biofilms in response to the treatment conditions. The extended incubation period was used since the antibiotic-resistant variants of *Pseudomonas aeruginosa* often have a delayed lag phase or slow growth phenotype^13^.

#### Replica plating method

For assessing whether electroceutical treatment was effective against TOB-resistant variants, three regions of interest (Supplementary Information, Fig. S1) were observed in our *in vitro* assay: (i) the regions primarily impacted by the TOB diffusing from the disks, (ii) the regions over the electrodes alone (primarily the anode in our case), and (iii) the overlapping regions influenced by both the electrode and TOB. Replica plating^13^ was used to assess whether all bacteria, including antibiotic-resistant variants^13,30^, were eliminated in these regions of interest (Supplementary Information).

Replica plating^13^ was performed on the prevention and treatment plates after *t* = 72 h and *t* = 96 h respectively by stamping the original plate onto Luria broth (LB) agar plates with and without 5 µg/mL TOB (Supplementary Information, Fig. S1), which is more than minimum inhibitory concentration, MIC, of TOB (1.5 µg/mL) for PA-Xen41^13^. Briefly, a sterile, cotton velveteen cloth (150 × 150 mm, Scienceware, Sigma-Aldrich, USA) aseptically wrapped and locked using an aluminum ring over a poly(vinyl) chloride replica plater (Scienceware, Sigma-Aldrich, USA) was used for stamping. In this replica plating assay, areas with no growth on the antibiotic-free replica plate show complete elimination of the bacteria in the biofilm (Supplementary Information, Fig. S1 shows both replica plates after 24 h of incubation at 37 °C and 5% CO_2_). Colonies which grow on both plates, i.e. with and without antibiotic, are TOB-resistant variants. All the replica plates were observed through IVIS imaging.

### Statistical analysis

All experiments to compare biofilm viability and luciferase activity between incubator and RT conditions were in triplicates. For multiple comparisons (i.e., between the anode, cathode, and control) with the data, at each time point, *t*, one-way ANOVA test with post hoc Tukey’s test was performed for determination of statistical significance (p ≤ 0.01). Statistics were performed on log transformed CFU data using MATLAB. The in-built function “anova1” was used to determine group means and variance while the in-built “multcompare” was used for post hoc test. The functional parameters ‘Alpha’ and ‘CType’ were set to 0.01 and ‘tukey-kramer’ respectively, corresponding to the significance level and test type.

## Results and Discussion

### Effects of electroceutical treatment on PA -Xen41 biofilm

Wound healing is delayed when the wound bed temperature decreases below normal core body temperature (37° C) or rises above 42° C^31^. Notably, the wound bed temperatures have been reported in a range of 25.3° C − 37.3° C with a mean of ~32.6° C^32,33^. Furthermore, biofilm growth and structure are known to be affected by environmental conditions^34^; however, the effect of changing ambient conditions such as temperature or the incubator environment on the electrochemical treatment of PA lawn biofilms has not been previously studied or reported. Therefore, lawn biofilms of PA-Xen41 (Fig. 1a) were subjected to electroceutical treatment for 24 h both at room temperature (RT) and in the incubator (37 °C and 5% CO_2_) to evaluate electroceutical treatment efficacy over the broad range of clinically relevant conditions^7^. At both RT and in the incubator, after *t* = 48 h (i.e., 24 h after treatment was turned off), a ~4-log decrease (p < 0.01) in biofilm viability (colony-forming unit per cm^2^, CFU/cm^2^) was observed at the anode compared to control (Fig. 1b,c). A difference of ~3-log (p < 0.01) was also observed at the anode compared to the cathode (Fig. 1b,c) at both RT and in the incubator. However, no significant reduction in biofilm viability was observed (Fig. 1b,c) at the cathode compared to control at any time points (p > 0.05).

**Figure 2:**
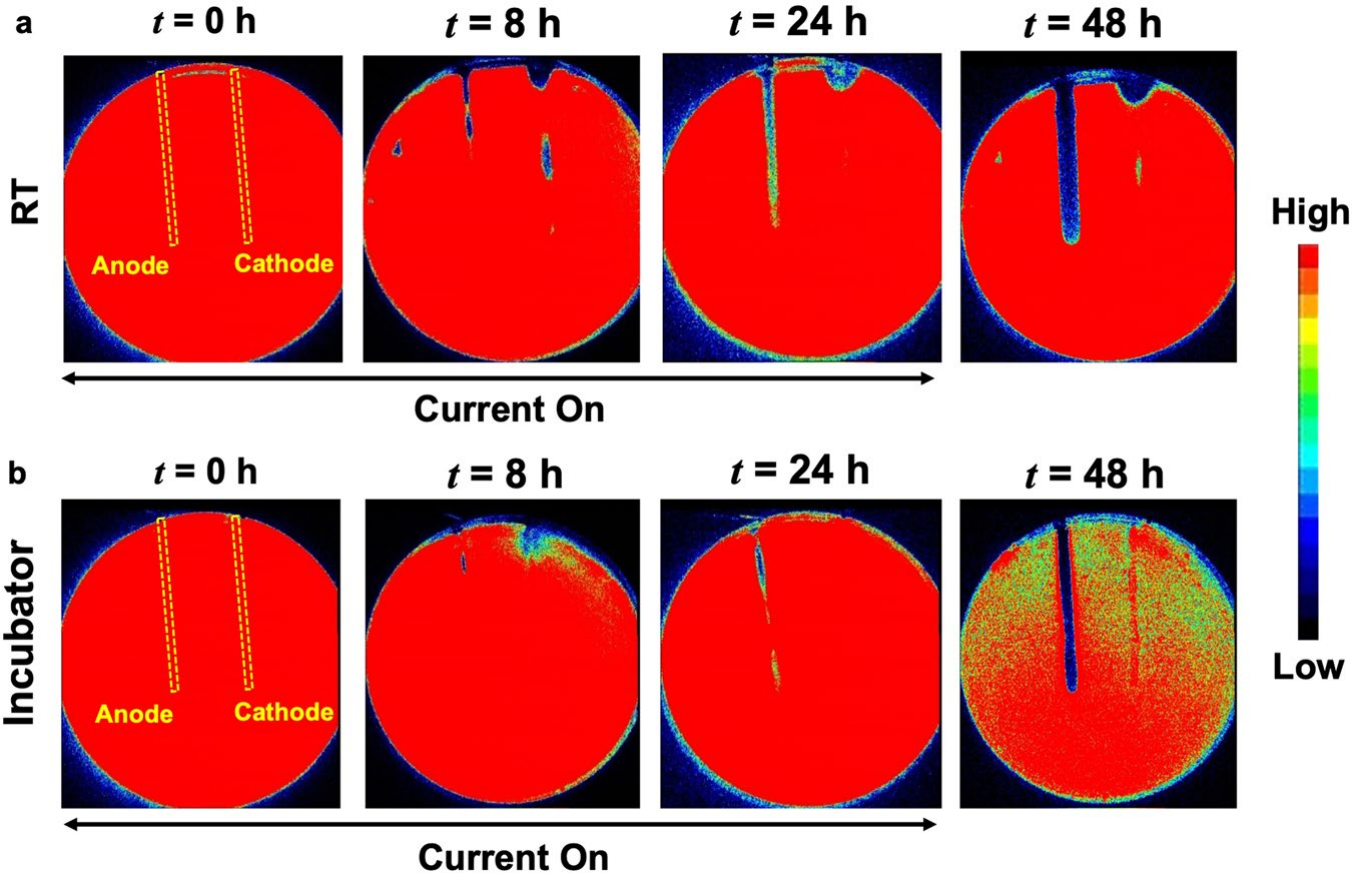
Bacterial luciferase activity maps as IVIS images at **(a)** RT and **(b)** in the incubator (37 °C and 5% CO_2_). At the anode, the zone of inhibition grew steadily over the course of electroceutical treatment i.e., when the current was ‘on’, and continued after the treatment ended (i.e., after the current was turned off) for both at RT and incubator conditions. At the cathode, a visibly larger area of inhibition (dark blue or black according to color map) was observed for the biofilm treated at RT compared to the incubator conditions. Biofilm killing and inactivation of metabolic activity is represented by the color map on the right with black/dark blue areas denoting biofilm killing.

At the anode, the zone of inhibition (i.e., zones of low or no metabolic activity) increased in width over the electrodes during the electrochemical treatment both at RT and in the incubator^26^ (Fig. 2). HOCl has been previously identified^26^ as the bactericidal agent generated at the anode. The electrochemical characterization of the *in vitro* set-up is shown in Supplementary Information, Figs. S2–S5. Notably, the zones of inhibition were spatially non-uniform over the cathode (Fig. 2a) for the biofilm treated at RT. The observed non-uniformities for bactericidal effects over the cathode compared to the anode are likely due to local conditions, including possible gas bubble entrapment over electrodes (Supplementary Information, Fig. S3), non-uniformities in the agar gels^26^. The exact mechanistic details for the observed differences are beyond the scope of this work and remain an active topic of investigation for future studies. Nevertheless, the zones of inhibition over the cathode were visibly larger for the biofilm treated at RT than that treated in the incubator for *t* = 8 h, 24 h, and 48 h.

It is worthwhile to note that the CFU/cm^2^ data (Fig. 1b,c) only presents a snapshot of the effects of electroceutical treatment over the punch biopsy area and is a ‘region of interest’ result that only captures bacterial eradication if the sampled area was directly impacted electrochemically. Eradication of the biofilm observed at RT was not spatially uniform over the cathode (Fig. 2a), and it is important to note that the biopsy punches (3 mm diameter) were consistently taken at the middle of the electrode, therefore CFU/cm^2^ might not reflect a complete picture of the bactericidal effects at the cathode due to the electrochemical treatment. However, the electrochemical treatment was effective in the eradication of PA-Xen41 lawn biofilm over the anode (~4-log decrease in biofilm viability) at both RT and incubator conditions.

After 24 h of electroceutical treatment, the pH reported earlier^26^ at cathode and anode were 8.40 ± 0.1 and 6.71 ± 0.1 respectively for the biofilm treated in the incubator, which agrees with the results reported in Table 1. It was previously reported^26^ that OH^−^ is generated at the cathode, leading to the measured increase in the pH (Table 1) at both RT and in the incubator compared to control. For the biofilm treated at RT, the pH at cathode was 11.46 ± 0.26 at *t* = 8 h and 9.82 ± 0.26 at *t* = 24 h, while for the biofilm treated electroceutically at incubator conditions, the pH at the cathode was measured to be 9.33 ± 0.19 at *t* = 8 h and 8.48 ± 0.18 at *t* = 24 h. These observations indicate that the higher pH and lower ambient temperature^34–36^ possibly leads to the visibly larger zones of inhibition over the cathode for the biofilm treated at RT (Fig. 2a). Finally, the differences in pH at cathode between RT and the incubator conditions are likely due to the carbonate buffering of CO_2_ in the incubator.

**Table 1:** Average pH measurements with standard deviations.

Time, *t*	Room Temperature (RT)			Incubator (T = 37 °C; 5% CO_2_)		
	Anode	Cathode	Control	Anode	Cathode	Control
0 h	7.31 ± 0.03			7.31 ± 0.03		
8 h	7.21 ± 0.28	11.46 ± 0.26	7.18 ± 0.19	6.46 ± 0.45	9.33 ± 0.19	6.78 ± 0.10
24 h	6.21 ± 0.90	9.82 ± 0.26	7.21 ± 0.21	6.86 ± 0.24	8.48 ± 0.18	6.98 ± 0.18

### Ultrastructure changes in the biofilm architecture under electrochemical treatment

**Figure 3:**
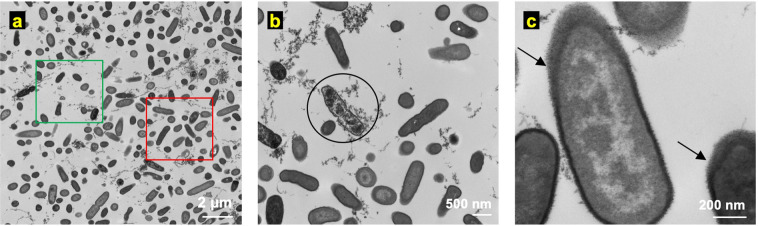
Ultrastructure images of PA-Xen41 lawn biofilm using transmission electron microscopy (TEM) at *t* = 0 h i.e., before the electroceutical treatment. **(a)** TEM image at lower magnification (6,800×) shows regions of higher cell density (red square) and interstitial ‘void’ spaces (green square). **(b)** Bacterial cells (circled) with expelled cellular content were also observed in the control samples. **(c)** At higher magnification (68,000×), bacterial cells in control samples showed lipopolysaccharide (LPS) around the cell (black arrows) whereas the interior of the cell had two distinct electron-dense regions—nucleoid (light) and cytoplasm (dark).

Transmission Electron Microscopy (TEM) displays high-resolution images of cellular ultrastructure i.e., the sub-cellular structure and the structure of the cell envelope, as compared to other imaging methods such as scanning electron microscopy that are limited to imaging the topography of microbial communities leaving the underlying sub-cellular structure unexamined^37^, or optical microscopy which is useful for features with characteristic length scales of a few hundred nanometers and larger. Therefore, ultrastructure changes in PA-Xen41 lawn biofilms were recorded using a TEM at three time points (*t* = 0, 8, and 24 h) for the electroceutical treatment in the incubator (T = 37 °C; 5% CO_2_). Figure 3 shows the TEM images before the electroceutical treatment started (i.e., *t* = 0 h). The TEM images reported here showed non-uniformly packed cells (red square in Fig. 3a) with interstitial ‘void’ spaces (green square in Fig. 3a) similar to images reported previously^37^. Bacterial cells (examples marked by a circle in Fig. 3b) with expelled cellular content, possibly DNA fibers with images showing structures similar to previously reported images^38^ (Fig. 3b), were also observed. Finally, normal or intact bacterial cells in the control sample showed two distinct electron densities (lighter and darker gray areas within the cells, Fig. 3c). Past ultrastructure imaging has shown that the cellular DNA could be concentrated in the light gray region which is represented as the low electron density region for TEM imaging^38^.

**Figure 4:**
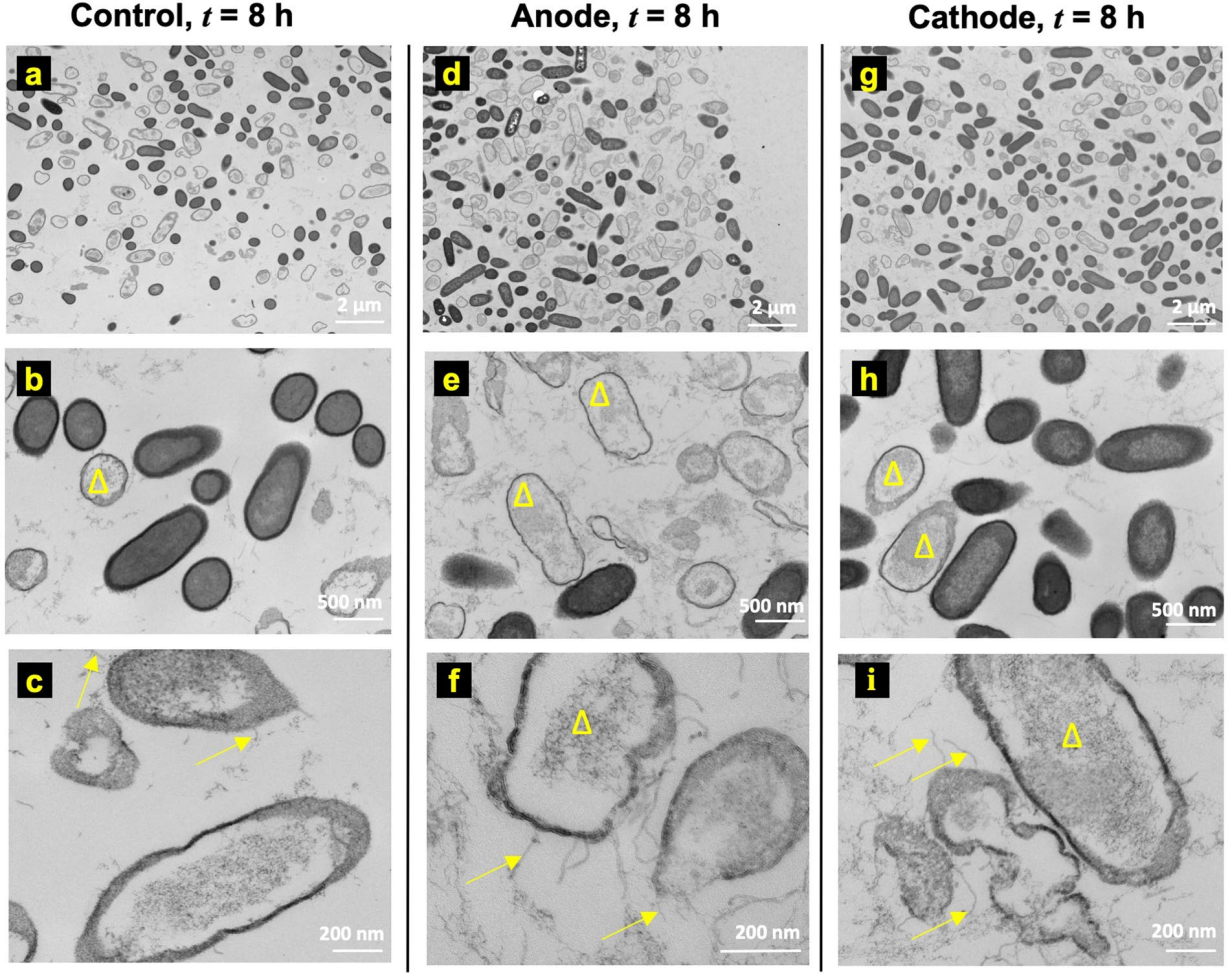
Ultrastructure observations at *t* = 8 h do not show any significant changes between **(a**–**c)** control, **(d**–**f)** anode, and **(g**–**i)** cathode. However, unidentified pili or fibril-like structures (yellow arrows in **c**, **f**, **i**) were observed in all cases on bacteria that appear less electron-dense compared to normal bacteria (**b**, **e**, and **h**) post-8 h electroceutical treatment (yellow triangles).

Figure 4 shows the ultrastructure images of the control (Fig. 4a–c), anode (Fig. 4d–f), and cathode (Fig. 4g–i) at *t* = 8 h after the treatment was started. The TEM images with the 2 µm scale (magnification: 6800×) do not show significant differences in cellular morphology between the control (Fig. 4a), anode (Fig. 4d), and cathode (Fig. 4g) regions. Notably, two different morphologies, one similar to normal bacteria as observed at *t* = 0 h and another where bacterial cells lacked cytoplasmic content (marked by yellow triangles), were observed in control (Fig. 4a–c), anode (Fig. 4d–f), and cathode (Fig. 4g–i) samples.

Note that Fig. 2b shows only small regions of reduced activity under IVIS over the anode at *t* = 8 h. The differences between this localized region of bacterial inhibition observed using IVIS for the incubator (37 °C) biofilms compared to the observed ultrastructure could be attributed to the fact that the TEM images represent a small area or region of interest imaging, with the field of view length scales on the order of 100 μm or less, on the samples consistently taken over the center of the electrodes using a 3 mm diameter punch (see methods section), and therefore, cannot capture the larger area (~3 mm × 8 cm) over the whole anode imaged using IVIS technology.

Interestingly, the pili or fibril-like structures (Fig. 4c,f,i) were observed on several bacterial cells for control, anode, and cathode samples that were less electron-dense compared to normal cells (Fig. 4b,e,h). These pili or fibril-like structures are marked in Fig. 4c,f,i by yellow arrows. Moreover, such pili or fibril-like structures have not been reported previously for PA lawn biofilms and the appearance of these structures regardless of the electrochemical treatment shows that these may not be an artifact due to the treatment methodology used here. Further work beyond the scope of this paper is needed to assess, identify, and quantify these structures.

**Figure 5:**
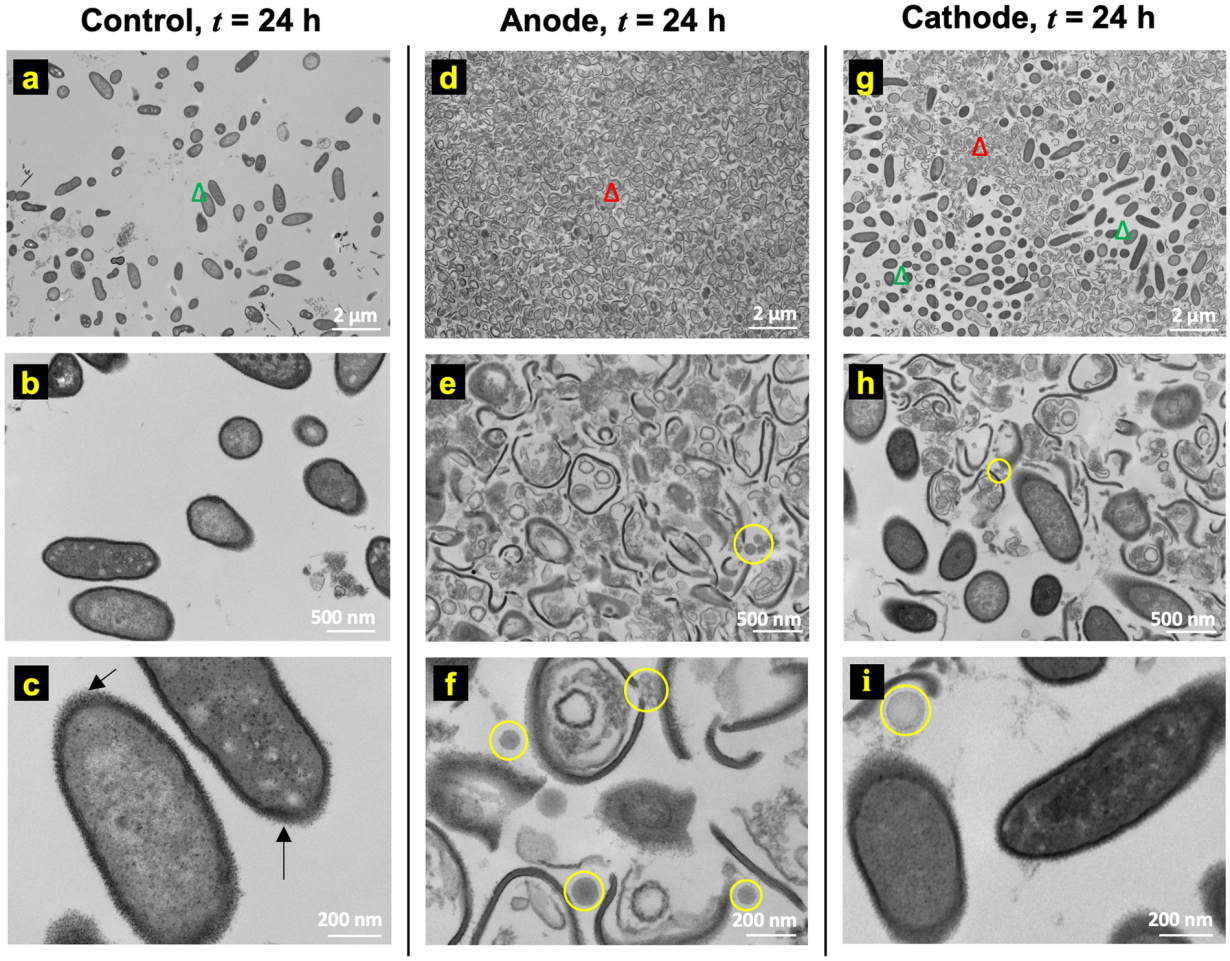
Ultrastructure images of the biofilm at *t* = 24 h on **(a**–**c)** control, **(d**–**f)** anode, and **(g**–**i)** cathode. The nucleoid and cytoplasm can also be distinguished respectively in less electron-dense (lighter) and comparatively higher electron-dense (darker) regions in the un-lysed or intact bacteria (green triangle) obtained for control (**a**–**c**), whereas solid black arrows (→) show lipopolysaccharide (LPS) around the bacteria (**c**). Comparatively, mostly cellular debris (red triangle) is seen on the anode (**d**–**f**) as opposed to intact bacteria (green triangle). On the cathode (**g**–**i**), a heterogeneous mixture of intact and ruptured bacteria was observed. Additional structures were observed that visually resemble membrane vesicles (MV) (yellow circles in **e**, **f**, **h**, and **i**) at both the anode and the cathode.

Figure 5 shows TEM images for *t* = 24 h after starting the electroceutical treatment. The control samples (i.e., with no electroceutical treatment) showed white spots in the interior of the cells (Fig. 5a–c). Past reports suggest that these spots may be granules of organic or inorganic material^38,39^ called inclusion bodies. Images for samples collected over the anode at *t* = 24 h show cell debris and significantly damaged bacterial cells with the cell envelope ruptured (Fig. 5d–f). The TEM images reflect the regions of near-complete inactivation of the bacteria in the IVIS images (Fig. 2b) and a ~4 log decrease observed in CFU/cm^2^ data due to bactericidal species produced at the anode. Electrochemically generated reactive chlorine species (RCS) such as HOCl produced at the anode (Supplementary Information, Fig. S5) is known to eradicate biofilm-associated bacteria^26^. HOCl is a powerful oxidant and most likely kills bacteria by simultaneously damaging multiple components in the cell with evidence suggesting that the inner membrane might be the site of fatal damage^22,40,41^. HOCl also oxidizes cytoplasmic enzymes, such as sulfhydryl enzymes, amino acids, inhibits cell growth, protein and DNA synthesis^22^. Furthermore, HOCl oxidizes components of outer membrane and periplasmic proteins^22,41^, and has also been implicated in oxidative unfolding and aggregation of bacterial proteins^42^. Ultrastructure images in Fig. 5d–f show significant bacterial cell damage at the anode, including ruptured cell envelope and denatured cytoplasmic content likely from the multi-factorial damage caused by the oxidative action of RCS.

In TEM images for samples collected over the cathode, both intact bacterial cells and damaged cells were observed. Compared to the anode, at *t* = 24 h, IVIS images do not show any biofilm inactivity (Fig. 2b) while the TEM images show both intact bacteria and damaged bacteria. Taken together, these results suggest that while some of the bacteria were killed on cathode (Fig. 5) yet the surviving bacterial population could still saturate the IVIS activity maps.

Over both the anode and the cathode, the lysed or damaged bacteria showed increased cellular debris as compared with the control samples. Notably, cell damage at both the anode and the cathode was also accompanied with the presence of other structures within the size range ~50–150 nm (highlighted by yellow circles). It has been reported previously that the size of membrane vesicles (MVs) range from ~50–150 nm in *Pseudomonas aeruginosa*^43^ biofilms. Notably, these MV-like structures were not observed in control samples at *t* = 0 h, 8 h or 24 h (Figs. 3–5) nor at the anode or the cathode at *t* = 0 h and 8 h time points (Fig. 5e,h,f,i).

### Electrochemical treatment of tobramycin-resistant variants

Tobramycin is an aminoglycoside antibiotic that is commonly used to resolve infections from gram-negative pathogens, such as PA^11,16,44^. Although antimicrobial resistance of PA biofilms is typically multi-factorial and still not fully understood^45^, nonetheless, the use of aminoglycosides like tobramycin has not only been linked to the development of resistance in PA biofilms by production of periplasmic glucans that can bind and sequester tobramycin before it reaches the target sites^19,46^ or to the expression of novel efflux pumps in the biofilms^47^, but in fact to the induction of the biofilm formation itself^48^.

**Figure 6:**
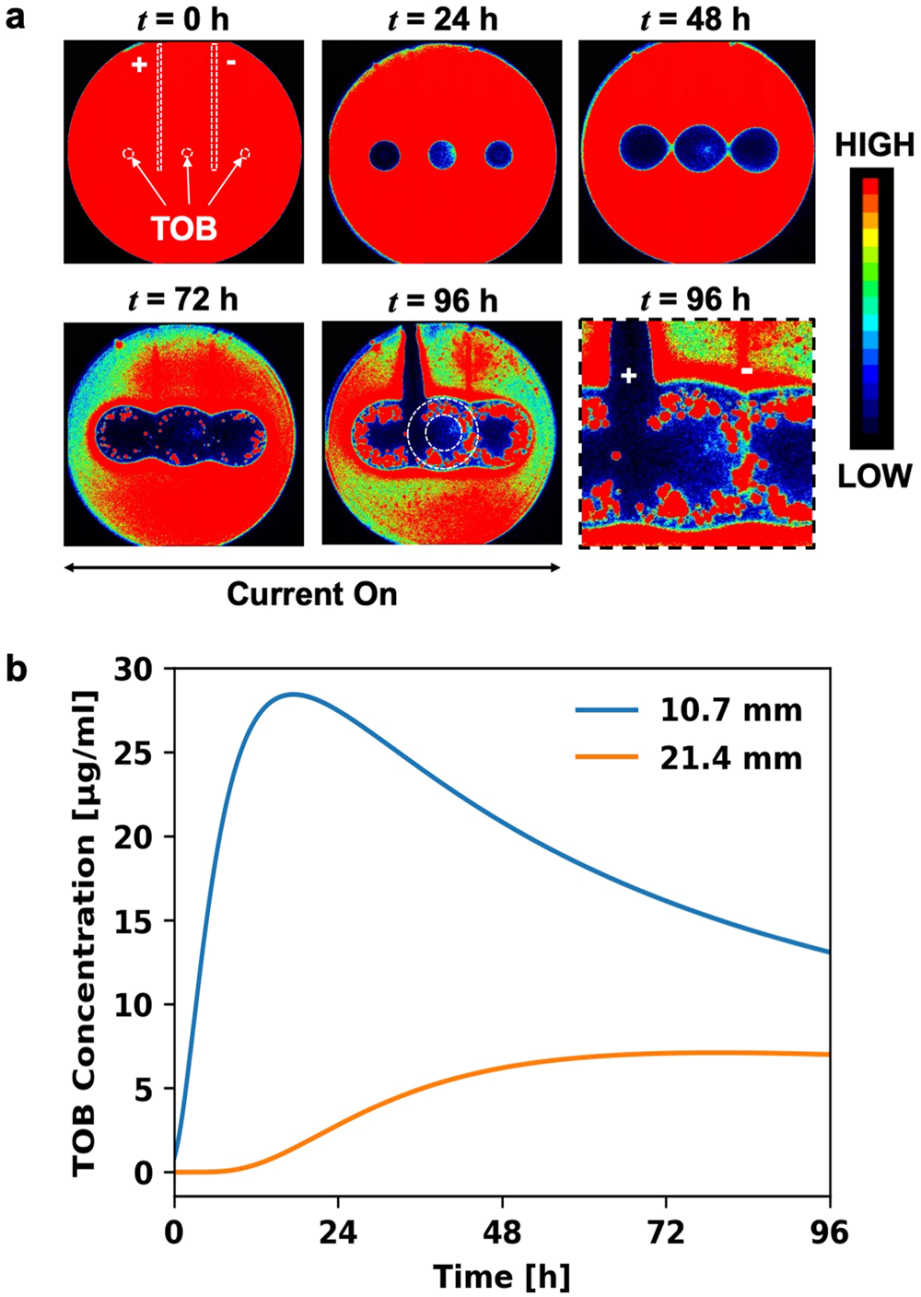
Treatment of antibiotic-resistant variants in PA-Xen41 lawn biofilm in the incubator (37 °C and 5% CO_2_). **(a)** The placement of TOB loaded sources i.e. paper disks, and electrodes is shown at *t* = 0 h. The electroceutical treatment was started at *t* = 72 h i.e., current was turned ‘on’ after the resistant variants emerged (red dots in the zones of inhibition around the TOB sources) and continued for the next 24 h. The resistant phenotypes present in the zones with estimated radii ranging between ~10.7 mm and ~21.4 mm from the center of each paper disk (dotted circles) were eliminated on the anode after 24 h of electroceutical treatment (at *t* = 96 h) while no such effect was observed over the cathode as determined by IVIS imaging. The last panel for IVIS imaging (bottom right) shows a zoomed in activity map at *t* = 96 h on anode (+) and cathode (−). **(b)** Tobramycin (TOB) concentrations were modeled as a function of time at radii ~10.7 mm and ~21.4 mm from a TOB-loaded (100 µg) disk. At *t* = 96 h, TOB concentrations were 13.34 μg/mL and 7.02 μg/mL at inner and the outer radii respectively.

In response to the *in vitro* tobramycin (TOB) treatment of lawn biofilms on agar, tobramycin-resistant variants emerge (Fig. 6a) in the clearance zone around the antibiotic source at approximately *t* = 72 h^13^. The electroceutical treatment was started after the TOB-resistant phenotypes were observed at *t* = 72 h in the clearance zone (Fig. 6a) and was carried out for the next 24 h (i.e., until *t* = 96 h). At *t* = 96 h, the tobramycin resistant colonies, which emerged between radius (*r*) ~10.7 mm and ~21.4 mm from the center of paper disks, were eradicated by the electroceutical treatment over the anode (Fig. 6a). In comparison, over the cathode, the TOB-resistant variants were still metabolically active (Fig. 6a) after 24 h of electroceutical treatment.

For antibiotic-resistant variants, MIC is higher than the MIC for the susceptible phenotype^13,49^. An analytical solution was used to estimate the TOB concentration, *c(r, t)*, where the resistant colonies were observed. The solution for the diffusion of antibiotic in an agar plate from a finite source, such as a paper disk, can be given by^27,29^:

1 $$c(r,\,t)=\frac{m}{{h}_{0}}\frac{1}{4\pi D(t+{t}_{o})}\exp \left(-\frac{{r}^{2}}{4D(t+{t}_{o})}\right),$$

where the diffusion coefficient of TOB in agar is *D* and was experimentally determined^50^ to be 3.84 × 10^−10^ m^2^/s, *m* is the mass of antibiotic added to the source, ${h}_{0}$ is the height of the agar, and *r* is the position coordinate relative to the center of a paper disk. Note that, in order to estimate TOB concentration, time (*t*) in Equation 1 is offset with a correction term *t*_*o*_ = ${r}_{o}^{2}/\text{8D}$ to account for the presence of a finite source, i.e., paper disk with radius, *r*_*o*_ = 3 mm, as reported previously^27^. Both the inner *radius* of the petri dish, 70 mm (inner diameter is 140 mm), and the center-to-center distance between the paper disks (35 mm) were >10 times the radius of the paper disks (3 mm) used, whereas the depth of agar layer was ~3.6 mm.

At *t* = 96 h, the TOB concentration (from Equation 1) was estimated to be ~13.34 μg/mL and ~7.02 μg/mL (Fig. 6b) at the inner and the outer radii enveloping tobramycin resistant colonies, i.e., at 10.7 mm and 21.4 mm, respectively. Notably, these TOB concentrations were greater than 5 μg/mL, i.e., the TOB concentration previously used^13^ to screen for resistant variants (see section on replica plating methods in Supplementary Information) and the MIC for the susceptible phenotype^13,49^. The TOB-resistant variants (active in the estimated TOB concentration range of 7.02–13.34 μg/mL) were eradicated on the anode after 24 h of electroceutical treatment (Supplementary Information, Fig. S1a), while no visible effect on the resistant variant colonies was observed on the cathode (Fig. 6a) based on IVIS imaging.

### Preventing emergence of antibiotic-resistant variants

**Figure 7:**
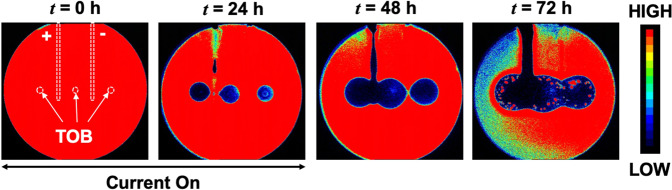
Preventing the emergence of the antibiotic resistant variants in the PA-Xen41 lawn biofilm. Electroceutical treatment was applied for 24 h along with the antibiotic treatment using a paper disk loaded with 100 μg TOB. Resistant PA-Xen41 colonies did not grow over the anode (*t* = 72 h) after combined treatment with tobramycin and the electroceutical.

Combined electroceutical and antibiotic treatment for PA-Xen41 lawn biofilm in the incubator (37 °C and 5% CO_2_) showed that, after 24 h of the electroceutical treatment, antibiotic-resistant variants did not emerge at *t* = 72 h (Fig. 7) over the anode. In contrast, these resistant variants were observed away from the anode and over the cathode (Fig. 7).

## Summary and Conclusions

Electroceutical treatment was compared both at RT and in the incubator using the time-lapse quantification of biofilm viability and bacterial luciferase activity maps of PA-Xen41 lawn biofilms. A ~4 log decrease (p < 0.01) in CFU/cm^2^ was observed at the anode compared to control at both RT and the incubator conditions at 48 h after the start of treatment.

TEM imaging of the ultrastructure of PA-Xen41 lawn biofilms under the electroceutical treatment in an incubator showed that the bacterial cell envelopes were damaged, likely due to the RCS generated at the anode, in samples taken above the anode at *t* = 24 h; while a mixture of un-damaged bacterial population and cellular debris was observed over the cathode at *t* = 24 h.

At the anode, electroceutical treatment eliminated the tobramycin-resistant phenotypes which appear after ~3 days of TOB treatment on the PA-Xen41 lawn biofilms; whereas no effect under IVIS imaging was discernible on the resistant phenotypes at the cathode. When the electroceutical treatment was combined with tobramycin treatment for the first 24 h, the emergence of tobramycin-resistant phenotypes was prevented at the anode.

To the authors’ knowledge, the effects of electroceutical treatment on the ultrastructure of lawn biofilms are reported for the first time. Furthermore, the electroceutical treatment as well as the prevention of tobramycin-resistant variants which may emerge during antibiotic treatment of lawn biofilms were also demonstrated. Finally, these observations provide new information on the electroceutical treatment of PA-Xen41 lawn biofilms that will help develop electroceuticals as possible treatment methodologies in the future.

## Supplementary information

Supplementary Information.
