# Supplementary material for: Ultrastructure imaging of Pseudomonas aeruginosa lawn biofilms and eradication of the tobramycin-resistant variants under in vitro electroceutical treatment
Source: Sci Rep. 2020 Jun 18;10:9879. doi: 10.1038/s41598-020-66823-y (PMC7303171; doi:10.1038/s41598-020-66823-y)
Supplement: Supplementary file 1 — Supplementary Information. [file 41598_2020_66823_MOESM1_ESM.docx]

**Ultrastructure imaging of *Pseudomonas aeruginosa* lawn biofilms and eradication of the tobramycin-resistant variants under *in vitro* electroceutical treatment**

**Varun Lochab^1^, Travis H. Jones^1^, Devendra H. Dusane^2^, Casey W. Peters^2^, Paul Stoodley^2, 3, 4^, Daniel J. Wozniak^2^, Vish V. Subramaniam^1^, and Shaurya Prakash^1, *^**

*^1^Department of Mechanical & Aerospace Engineering, The Ohio State University, Columbus, Ohio, USA*

*^2^Department of Microbial Infection & Immunity, The Ohio State University, Columbus, Ohio, USA*

*^3^Department of Orthopaedics, The Ohio State University, Columbus, Ohio, USA.*

*^4^National Centre for Advanced Tribology, Mechanical Engineering, University of Southampton, Southampton, UK.*

*^*^****Corresponding author****:*

*Shaurya Prakash*

*E347 Scott Lab, 201 W. 19th Avenue, Columbus, Ohio, 43210, United States*

*Phone: 614-688-4045*

*Email:* [*prakash.31@osu.edu*](mailto:prakash.31@osu.edu)

**SUPPLEMENTARY INFORMATION**

**Replica plating**

**Figure S1.** Activity maps from replica plates without TOB and with 5 μg/mL TOB to identify resistant phenotypes for (a) treatment (digital image at *t* = 96 h) and (b) prevention (digital image at *t* = 72 h). The regions impacted by the TOB diffusing from the disks (i), the region over the electrodes alone (ii, primarily the anode in our case), and the overlapping regions influenced by both the electrode and TOB (iii) are marked. The regions of no colonies (yellow arrows) on the plates with no TOB indicates that all bacteria in these regions were killed^1^, while the red arrows and green arrows show the growth of resistant variants and the zones of no growth on TOB plates, respectively. The paper disk positions in a 140 mm diameter petri dish are shown in the digital image (a) for treatment. The stamping area which was same for both cases is also delineated by the white, dashed circle.

To assess whether all bacteria had been killed in zones of biofilm clearance (Figure 6a) or inhibition (Figure 7), we used replica plating (Methods) onto two Luria broth (LB) agar plates, one with no antibiotics (TOB) and one containing TOB (5 µg/mL, which was above the MIC of the PA strain used). The regions of no colonies (yellow arrows, Figure S1) on the plates with no TOB indicates that all bacteria (resistant or susceptible population) in these regions were killed, not just suppressed. The plates with no TOB are not selective to resistant mutants, while only resistant variants selectively grow on the agar plates with 5 µg/mL TOB. As we had previously reported^1^, the growth of resistant variants was observed in a ring (red arrows, Figure S1) in region (i) around the antibiotic containing disks near the interface between the zone of inhibition and the outer zone where the biofilm lawn was unaffected. However, in addition to killing above the anode alone (Figure 6a, Figure 7), we observed gaps in the rings of resistant variants over the anode in the regions of overlap, i.e., region (iii, Figure S1), on the replica plates with TOB. Therefore, it was concluded that even resistant variants present in these regions were killed and/or suppressed from forming in this zone (green arrows) presumably by the combined action of the electrode and the antibiotic.

**Electrochemical characterization of *in vitro* system**

While past work developed an innovative *in vitro* model for infected tissue^2^, quantitative values for electrode potentials were not reported, which can be important for understanding the underlying mechanisms for electroceutical action. Therefore, an electrochemical characterization of the *in vitro* model (Fig. S2) was first conducted to measure the electric potential at the Ag electrodes. The open-circuit potentials for both anode and cathode were measured (Fig. S2b) simultaneously against Ag/AgCl reference electrodes (3M NaCl) (Bioanalytical Systems, Inc., BASi®) over 24 h after the system was switched on. A digital multi-meter (Keithley 2001) and open source potentiostat (Rodeostat, IO Rodeo) were used for measuring the potentials at the anode and cathode respectively (Fig. S2b), with both voltmeters providing voltage readings within ~ 1% of each other. The Ag/AgCl reference electrodes were positioned at 4 cm along the length of the 8 cm electrodes (i.e., the middle of both electrodes) and subsequently, the TSA was poured around the reference electrode and allowed to cool to RT to form the gel. All measurements for electrochemical characterization were done at RT and without the lawn biofilm, under the assumption that the presence of a lawn biofilm, with thickness ~ O (10^2^ µm), separated from the electrode by 3.6 mm thick layer of the agar will not change electrochemical reactions at the electrode surface.

**Figure S2. (a)** Schematic for electrochemical treatment of bioluminescent *Pseudomonas aeruginosa* (PA-Xen41) biofilm. After 24 h of electrochemical treatment, a layer of silver chloride (AgCl) was observed and confirmed in our previous work^2^ and OH^-^ was generated at the cathode. **(b)** The potential drop across the anode and the cathode were monitored in the absence of lawn biofilm according to the schematic shown for 24 h after the current flow was initiated. **(c)** The potential drop across the anode increased from 0.16 V at 0 h to about 5.34 V at 24 h, whereas the magnitude of the potential drop across the cathode decreases from ~ 1.6 V to ~ 0.5 V over the same period.

The potential drop across the anode increased from 0.16 V at *t* = 0 h to about 5.34 V at *t* = 24 h with most of the voltage drop occurring across the anode at 24 h (Fig. S2c); whereas, the magnitude of the potential drop across the cathode decreases from ~ 1.6 V to ~ 0.5 V (Fig. S2c) over the same time period. Previous observations showed that a ~ 45 μm thick layer of AgCl grows on the anode over 24 h whereas OH^-^ was generated at the cathode as visualized using the pH indicator dye^2^. The sharp fluctuations observed in the potential drop curve at the cathode (Fig. S2c) were likely due to the formation of hydrogen gas bubbles^2^ (Fig. S3).

**
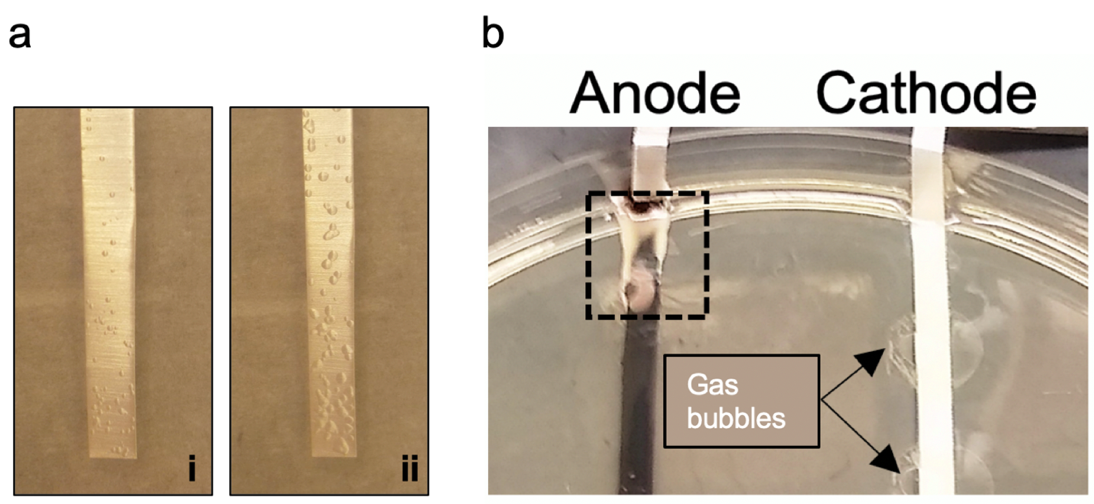
**

**Figure S3. (a)** Evolution of gas bubbles within *t*_i_ < 30 s (i) after the current flow was initiated, the bubbles coalesced and grew in 40 s (ii at *t*_i_ + 40s). **(b)** Digital image of electrodes at *t* = 8 h. On anode, non-heterogeneity in the deposition of silver chloride, variation in battery nominal voltage, uncontrolled electrode surface conditions might also lead to other electrochemical reactions (boxed) that are currently undetermined. On cathode, coalesced gas bubbles, likely from H_2_ gas, were observed both in plates with (not shown) and without lawn biofilms (shown), whereas the corresponding increase in the pH is shown in Fig. S4.

**Figure S4.** Phenol red pH indicator dye shows the spread of OH^-^ from the cathode at both RT and in the incubator (37° C and 5% CO_2_) within 4 h of the start of the treatment. pH was tracked (Table 1) over the course of 24 h after the current flow was started.

*Detection of free chlorine at the anode*

In our earlier work^2^, the chlorination of tyrosine was observed at the anode under electroceutical treatment indirectly indicating the production of RCS such as HOCl/OCl^-^ (free chlorine species^3^) at the electrode. However, detection of free chlorine species directly at the Ag electrode was previously not possible in the agar model with growth media since free chlorine species are powerful oxidants and known to react with organic matter^4, 5^, for example, the components of the growth media. Hence, the detection of HOCl/OCl^-^ as reported here was conducted in an aqueous solution of 5 g/L NaCl and 2.5 g/L K_2_HPO_4_ (equivalent to the concentration that is used for the preparation of TSA) made in deionized (DI) water, 18.2 MΩ·cm at 25°C.

First, using an Ag/AgCl reference electrode, the potential at the Ag electrode was held at 0.16 V (i.e., at the starting potential, Fig. S2c) for 1 h using Reference 600™ (Gamry Instruments). Within five minutes of maintaining the electrode potential, free chlorine was detected by a color change from colorless to magenta (Fig. S5) in the presence of N, N-diethyl-p-phenylenediamine or DPD (Hach DPD Free Chlorine Reagent Powder, ~70 mg per 5 mL as per vendor specifications), around the electrode. The current decreased exponentially with the formation of the resistive AgCl layer on the electrode as reported earlier^2^. After 1 h, the electrical potential was turned off and ultraviolet-visible (UV-vis) spectroscopy was performed using Evolution™ 300 UV-Vis Spectrophotometer (Thermo Fischer Scientific) on the aqueous samples collected at the Ag anode and from control (no electrical stimulation but containing 5 g/L NaCl and 2.5 g/L K_2_HPO_4_ aqueous solution with DPD). The samples were aliquoted to a 300 μL quartz micro cuvette with a pathlength of 10 mm (#14-958-133, Fischer Scientific). UV-vis spectroscopy also confirmed that the formation of AgCl layer was accompanied by free chlorine at the anode.

*
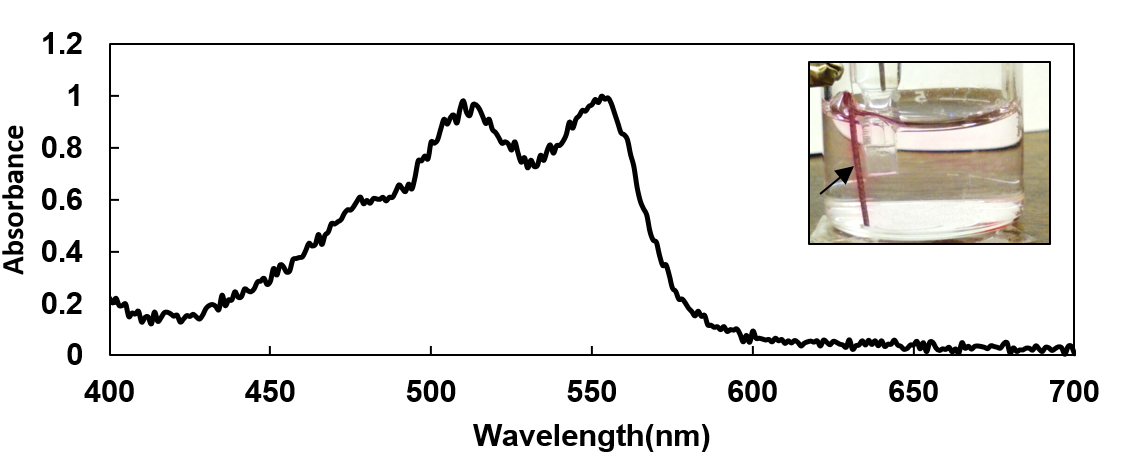
*

**Figure S5.** Ultraviolet-visible (UV-vis) spectroscopy was performed on a sample obtained after holding 0.16 V vs. Ag/AgCl reference electrode on an Ag electrode in an electrolyte with equivalent salt concentrations as TSB (Methods) for 1 h. N, N-diethyl-p-phenylenediamine (DPD) free chlorine reagent was used for the detection of free chlorine (magenta color around the electrode). A magenta hue is produced by the DPD reagent after a reaction with free chlorine (Inset). The presence of free chlorine at the anode was confirmed by the UV-vis spectra, which shows absorbance in blue-green region (400-600 nm) of the visible spectrum. The control (5 g/L NaCl and 2.5 g/L K_2_HPO_4_ aqueous solution with DPD) was used as the baseline.

**REFERENCES**

1. Dusane, D. *et al.* Complete killing of agar lawn biofilms by systematic spacing of antibiotic-loaded calcium sulfate beads. *Materials* **12**, doi:10.3390/ma12244052 (2019).

2. Dusane, D. H. *et al.* Electroceutical treatment of *Pseudomonas aeruginosa* biofilms. *Scientific Reports* **9**, 2008, doi:10.1038/s41598-018-37891-y (2019).

3. Jović, M. *et al.* Electrochemical detection of free chlorine at inkjet printed silver electrodes. *JEAC Journal of Electroanalytical Chemistry* **756**, 171-178 (2015).

4. Gray, M. J., Wholey, W. & Jakob, U. Bacterial responses to reactive chlorine species. *Annual Review of Microbiology* **67**, 141-160, doi:10.1146/annurev-micro-102912-142520 (2013).

5. Sedlak, D. L. & von Gunten, U. The chlorine dilemma. *Science* **331**, 42, doi:10.1126/science.1196397 (2011).
